# Investigating the Spectroscopy of the Gas Phase Guanine–Cytosine Pair: Keto versus Enol Configurations

**DOI:** 10.1021/acs.jpclett.3c02073

**Published:** 2023-09-28

**Authors:** Giacomo Botti, Michele Ceotto, Riccardo Conte

**Affiliations:** Dipartimento di Chimica, Università degli Studi di Milano, Via Golgi 19, 20133 Milano, Italy

## Abstract

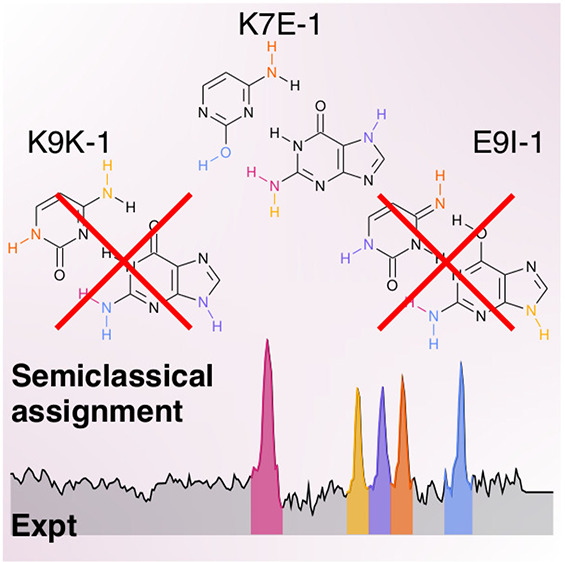

We report on a vibrational study of the guanine–cytosine dimer tautomers using state-of-the-art quasiclassical trajectory and semiclassical vibrational spectroscopy. The latter includes possible quantum mechanical effects. Through an accurate comparison to the experimental spectra, we are able to shine a light on the hydrogen bond network of one of the main subunits of DNA and put the experimental assignment on a solid footing. Our calculations corroborate the experimental conclusion that the global minimum Watson-and-Crick structure is not detected in the spectra, and there is no evidence of tunnel-effect-based double proton hopping. Our accurate assignment of the spectral features may also serve as a basis for the development of precise force fields to study the guanine–cytosine dimer.

Deoxyribonucleic acid (DNA) is a very important biopolymer because it stores cell genetic information. Its duplication process heavily relies on the complementarity of the nucleobases in forming hydrogen bonds. On the one hand, the nature of the hydrogen bond allows the DNA molecule to open and close the double helix, so that the genetic information can be duplicated by means of the DNA polymerase. On the other hand, this flexibility allows for the formation of other tertiary structures, such as B-DNA, A-DNA, or even triple-stranded DNA.^1^ Different base clusters are also possible, such as the Hoogsteen pairs and G-quadruplexes.^2,3^ Given the presence of keto and amino groups, nucleobases present a great number of tautomers, a characteristic that can induce mismatches during the replication processes.^4^ Even if ribonucleic acid (RNA) is the nucleic acid most affected by non-canonical pairing,^5^ the nucleobase pair with the highest number of stable tautomers is guanine–cytosine (GC), which is in common with DNA. Therefore, we will focus on this pair.

One of the proposed mutagenic mechanisms is initiated by a proton hopping event, in which the two hydrogen atoms involved in the supramolecular bonding hop from one base to the other. As a consequence, new tautomers are generated, and these are then mistakenly paired during DNA reproduction. However, the debate around the mechanism of this proton hopping is still open.^6−11^ On the one hand, the double proton hopping process is thermally improbable, and products are too short-lived to have any biological impact, even if this aspect does not exclude that the double proton hopping could happen as a defense mechanism, in a deactivation pathway.^10,12,13^ On the other hand, the process could occur via tunneling, and this would be fast enough to make the product short life irrelevant.^8^ If this is the case, the hopping tautomer could enter the polymerase λ pocket and be incorporated erroneously.^7^ The discussion about tunneling in DNA bases is still open,^9,14^ and at the moment, this hypothesis has not been confirmed. More specifically, at the moment of our writing, no clear evidence of double proton hopping has been found, but the continuous development of fast and ultrafast techniques might provide the experimentalists with the needed tools to catch this phenomenon if really present.

To unravel the DNA structural and mechanical peculiarities, it is necessary to understand the equilibrium and, more importantly, the dynamical properties of the pairs of nucleobases and focus on the hydrogen bond network. We think that understanding the dynamics of the hydrogen couple confined between the two nucleobases is pivotal to develop an accurate model for *in vivo* DNA stability and also for base tautomerization and mismatch, even more if quantum effects are to be included.

To this aim, we employ vibrational spectroscopy because the effects of hydrogen bonding can be easily detected through it, and non-trivial information can be extracted when the vibrational spectra of hydrogen-bonded species are compared to the corresponding non-bonded species.^15−28^ Therefore, the experimental vibrational spectra of nucleobase dimers are interesting and well-known in the gas phase, where the hydrogen bond contribution to the pair formation and stability can be isolated from the π stacking and hydrophobic interaction. The hydrogen-bonded structure is observed only in the gas phase, because in aqueous solution, the bases prefer the stacked disposition.^29,30^ The vibrational spectra could also help to clarify if any proton hopping is taking place, in particular in the guanine–cytosine pair, by inspecting if specific features of the proton-hopped tautomer are present in the spectra.^29,31−36^ The main current limitation of these experiments is the absence of a solid and thorough assignment of the main spectral features, which would allow one to clearly identify the corresponding tautomer. Specifically, the experimental spectrum of the GC dimer is currently assigned to a high-energy tautomer (named K7E-1), instead of the expected global minimum tautomer, which is the Watson and Crick (WC or K9K-1) structure.^29,37^ This spectrum has been obtained by infrared (IR)–ultraviolet (UV) hole burning spectroscopy on a mixture of laser-desorbed guanine and cytosine.^32,33^

The interest in comparing our simulations to the experimental findings is 2-fold. The first goal is to check whether the WC structure is really not present in the experimental spectrum. Nir et al. based their assignment on a scaled harmonic approach, which is often a non-reliable and *ad hoc* technique not able to include quantum effects, and the isolated base computational setup spectra, which do not account for intermolecular effects. Therefore, it is possible that a precise quantum assignment of the WC tautomer spectrum matches the experimental spectrum, demonstrating the presence of relevant quantum effects. However, if conversely the absence of the WC tautomer in the spectrum is confirmed, then this fact would support the hypothesis that the WC excited state lifetime is unexpectedly short.^29,32,38^

Then, we want to see whether the vibrational spectrum may reveal the fingerprints of quantum effects related to the double proton hopping mechanism. There are two main ways by which we can point out this phenomenon: We can simulate the molecular species, which would be the result of the double proton hopping starting from the WC tautomer, i.e., an enolic form labeled as E9K-1, and check if it is present in the experimental spectrum; furthermore, it is possible to look for differences in the spectral features between calculated WC tautomer spectra obtained by means of a theoretical method able to point out quantum effects and another method unable to do that. In general, an accurate assignment of the experimental spectrum by means of a refined theoretical technique will be of great help to build force field models to be employed in the study of the GC dimer.

Current computational and theoretical vibrational studies of DNA bases are limited to static (harmonic-like) approximations or low-dimensional models as a result of the system intrinsic complexity.^32,34,39,40^ These approximations fall short when dealing with hydrogen-bonded systems,^28^ and a higher accuracy is necessary if one wants to assign nucleobase pair experimental spectra, where there are a pletora of tautomers.^31−33^ To overcome these limitations, we employ semiclassical dynamics, an active area of research,^41,42^ and specifically the divide-and-conquer semiclassical initial value representation (DC SCIVR) method to compute the vibrational power spectra of the guanine–cytosine dimer.^43^ DC SCIVR is an acknowledged method, capable of accounting for anharmonicity and reproducing quantum effects, such as zero-point energy (zpe), overtones, and combination bands, using a single classical trajectory.^44−46^ This allows us to limit the computational effort even when investigating the 29 atom guanine–cytosine pair. DC SCIVR has already been applied with success to the vibrational study of isolated and solvated nucleobases and to other nucleotide-based macromolecules.^3,24,47,48^ The goal of these DC SCIVR spectra is to assign the experimental features on a solid footing and clear the open issues about both the structure and stability of the nucleobase pairs.

The time-averaged semiclassical initial value representation (TA SCIVR)^49−52^ is a quantum approximate method, which can be applied to spectroscopy calculations of moderate dimension systems. In TA SCIVR, the vibrational power spectrum *I*(*E*) can be computed as
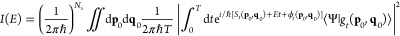
1where *E* is the vibrational energy, *N*_υ_ is the number of vibrational degrees of freedom, (**p**_0_,**q**_0_) are the starting conditions, *T* is the total simulation time, *S*_*t*_(**p**_0_,**q**_0_) and ϕ_*t*_(**p**_0_,**q**_0_) are the instantaneous classical action and phase of the Herman–Kluk pre-exponential factor,^53^ respectively, and ⟨Ψ|*g*_*t*_(**p**_0_,**q**_0_)⟩ is the quantum overlap between an arbitrary reference state |Ψ⟩ and a coherent state evolved for a time *t* (|*g*_*t*_(**p**_0_,**q**_0_)⟩). Additional details can be found in the Supporting Information.

The multiple coherent semiclassical initial value representation (MC SCIVR) was introduced to adapt the TA SCIVR method to on-the-fly calculations.^54−57^ MC SCIVR is based on two pillars: first, a single and tailored semiclassical trajectory at the exact quantum energy is able to fully describe the quantum state;^58^ second, it is possible to increase the vibrational signal collected by this single trajectory by defining the reference state |Ψ⟩ by means of an appropriate combination of two coherent states. Therefore, when MC SCIVR is employed, a single tailored trajectory is enough to compute an accurate vibrational power spectrum.^57^

However, when dealing with large systems, the curse of dimensionality sets in. This is a decrease in the signal-to-noise ratio of the power spectrum, caused by the superposition integral in eq 1, which goes to zero when the number of degrees of freedom increases. To tackle this curse, the divide and conquer (DC) technique was developed.^43,59^ In DC SCIVR the semiclassical power spectrum is computed on a subspace of the full dimensional space, while the trajectory is still evolved in full dimensionality and partly able to recollect interactions between modes belonging to different subspaces. Additional details on these semiclassical approaches, useful to help to replicate results, can be found in the Supporting Information.

When a semiclassical calculation is performed, it can also be useful to compute the classical velocity autocorrelation spectra employing the same MC-SCIVR classical trajectory as a term of comparison, thus performing a quasiclassical trajectory (QCT) calculation. The QCT spectrum of the *j*th vibrational normal mode is evaluated as^60,61^
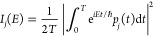
2where *T* is the total classical trajectory time, *E* is the energy, and *p*_*j*_(*t*) is the linear momentum of the *j*th vibrational normal mode at time *t*. We perform an *ab initio* “on-the-fly” Cartesian evolution of the dynamics with Cartesian coordinates and momenta transformed into normal mode coordinates and momenta at each time step along the trajectory. The QCT spectrum is able to include the potential energy surface anharmonicity, but it cannot collect any quantum effect. Nonetheless, it provides affordable and important data and has been applied with success to several systems.^15,28,60^

The conformational landscape of the GC pair is quite varied because it depends upon the tautomeric forms of both guanine and cytosine and their relative positions, i.e., the hydrogen bond network. Therefore, we adopt the nomenclature established in the experimental literature.^32,33^ According to this nomenclature, the GC pair tautomer is identified by indicating (i) the guanine tautomer, which can be either enol (E) or keto (K), (ii) the position of imidazolic hydrogen of guanine, which is either on nitrogen 7 or 9, (iii) the tautomer of cytosine, which is keto (K), enol (E), or imino- (I), and (iv) a number related to the relative energy of the hydrogen bond network. In this nomenclature, the Watson and Crick canonical tautomer is named K9K-1.

We start by studying the conformational landscape of the four tautomers lying lower in energy, reported in Figure 1. K9K-1 is the canonical and most stable tautomer, i.e., the WC tautomer. Then, we consider the K7E-1 tautomer because the literature suggests its presence in the experimental spectra. Furthermore, we focus on the K9E-1 tautomer because it is indistinguishable from K7E-1 when looking at the high harmonic frequencies, and it is more similar to K9K-1. Finally, we also consider the E9I-1 tautomer because it is formed upon double proton hopping starting from K9K-1, and the presence of E9I-1, even in traces, would suggest the presence of a fast-proceeding double proton hopping mechanism, resulting in a stable tautomeric form.^7−10,12,32^

**Figure 1:**
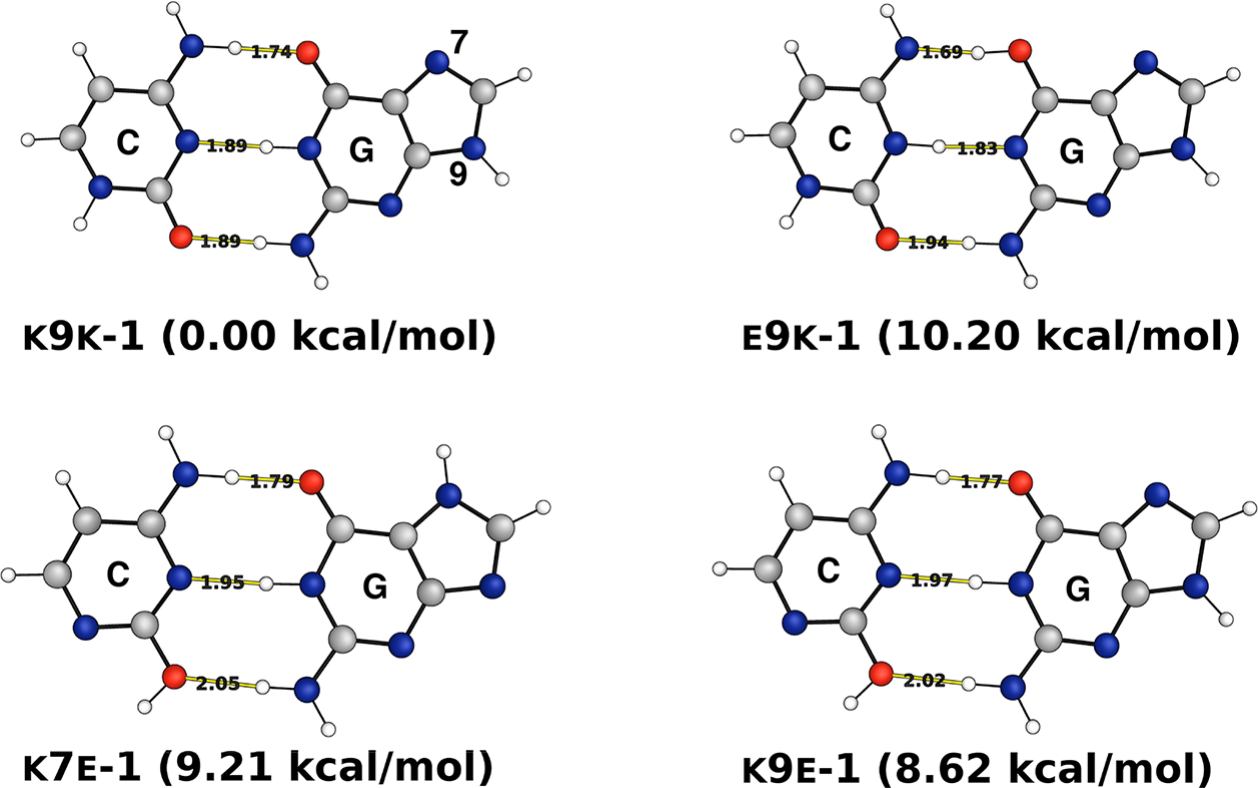
Equilibrium geometries and energies (kcal mol^–1^) of the four investigated GC tautomers, at the DFT-D/B3LYP def2-TZVP level of theory. The hydrogen bonds are indicated by the yellow lines, with distances in Å.

We optimize all of the anticipated tautomers at the DFT-D/B3LYP level of theory with the def2-TZVP basis set,^62^ where DFT-D stands for the density functional theory (DFT) with Grimme’s empirical dispersions.^63^ The equilibrium geometries with relative energies are listed in Figure 1. The complete energy analysis is reported in Table S2 of the Supporting Information. The relative energies are in good agreement with those reported in the literature, confirming the WC tautomer (i.e., K9K-1) as the global minimum of the hydrogen-bonded structure of the GC pair. These four conformers present very different energies, even if the hydrogen bond structure is quite similar.

Moving to spectroscopy, our reference experiments are those collected by de Vries et al. using the hole burning IR–UV spectroscopy in a time-of-flight (TOF) spectrometer.^29^ The experimental spectra were assigned using the spectra of the isolated bases and scaled harmonic frequencies at the RI-MP2 TZVPP *ab initio* level of theory.^29,32,33^ The spectrum obtained by laser ablation of guanine and cytosine was initially assigned as either K7E-1 or K9E-1.^32,33^ Upon investigation of the low-frequency region, it was concluded that the compound responsible for the spectrum was K7E-1, given the position of the N^7^H bending signal. The experimental assignment is summarized in Table S3 of the Supporting Information.

In our simulations, we start calculating the QCT power spectra of each tautomer, which we report in Figure 2. QCT spectra are obtained by means of eq 2 runs based on a 25 000 au trajectory at the DFT-D/B3LYP def2-TZVP level of theory. The focus is on modes above 3300 cm^–1^ for each tautomer. Furthermore, the QCT values are compared to the harmonic frequencies in Table S4 of the Supporting Information to appreciate the level of anharmonicity of the system. In the high-frequency region, the QCT power spectra of K9K-1 and E9I-1 reported on the upper part of Figure 2 are different, mainly in the guanine NH_2_ stretch. This suggests that the main difference in this frequency region is due to the O···HNH hydrogen bond on the guanine NH_2_ group. Given that QCT, in the absence of relevant quantum effects, is generally a pretty accurate method, this feature provides a way to confirm or deny the presence of E9I-1 in K9K-1 gas phase spectra. Conversely, in the lower part of Figure 2, the QCT power spectra of K7E-1 and K9E-1 in the same high-frequency region are basically indistinguishable from each other within the unavoidable peak width originating from the finite-time Fourier transform.

**Figure 2:**
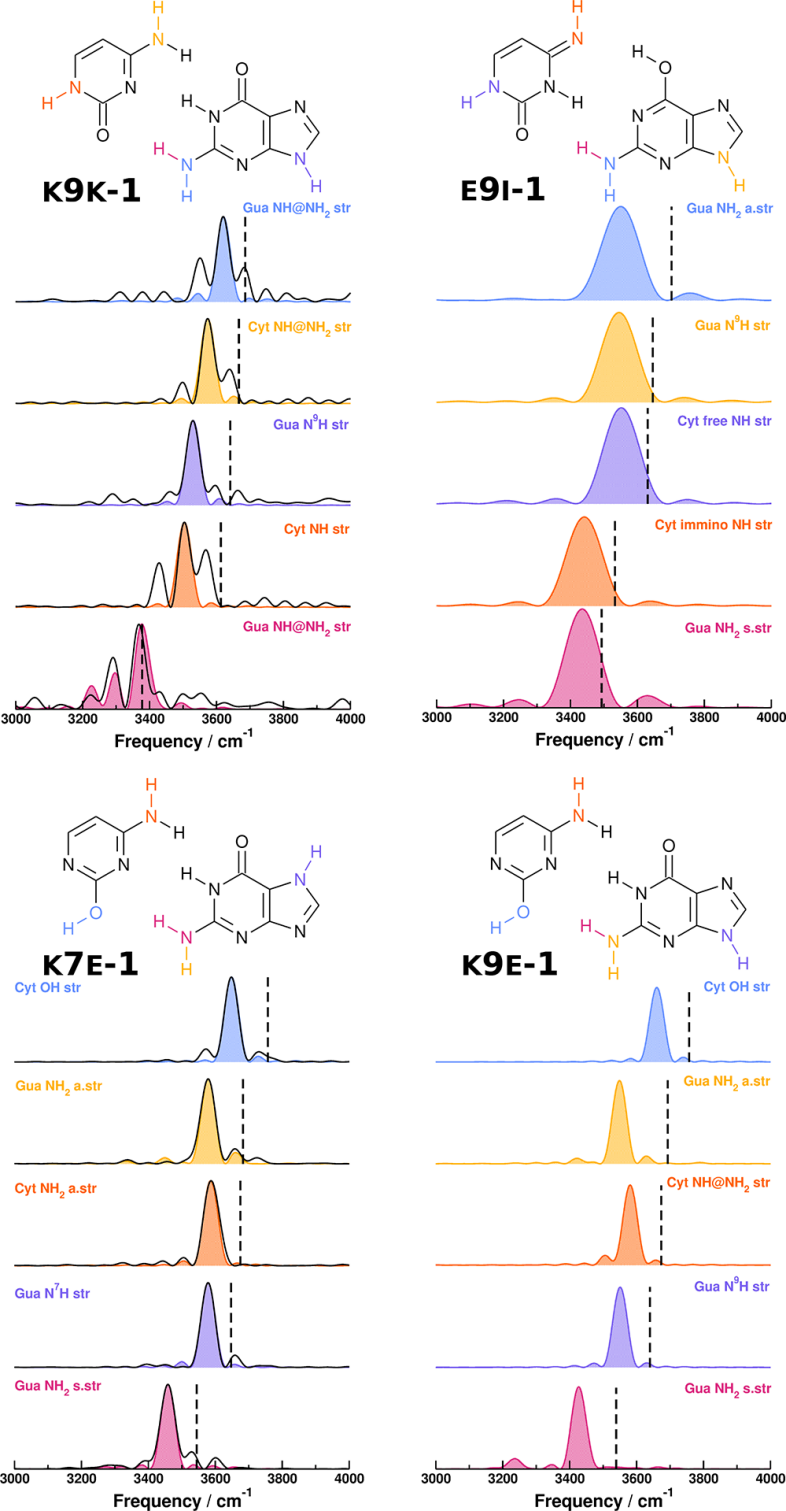
Power spectra in the frequency region above 3300 cm^–1^ for the four tautomers, obtained with QCT on a 25 000 au long trajectory, starting from harmonic conditions at the DFT-D/B3LYP def2-TZVP level of theory. The harmonic frequencies are reported as black dashed lines. For K9K-1 and K7E-1 the DC-SCIVR spectra are reported as black solid lines. See Table S4 of the Supporting Information and Table 1 for the numerical values.

As a comparison to these QCT results, we employed DC SCIVR for K9K-1 and K7E-1. We do not consider for DC SCIVR simulations the E9I-1 tautomer because it is populated only at high energies. Instead, we still consider the K7E-1 tautomer because it is reported as the main tautomer in experimental spectra, even if K9K-1 and K9E-1 are lower in energy.^29,32−34,37^ The DC SCIVR spectra are shown in Figure 2 as black solid lines, and they confirm the QCT spectra values.

The only way the experimentalists had to obtain a vibrational spectrum related to K9K-1 was to lock the tautomer by alkylation, thus collecting the spectrum of *ethyl*-K9-*methyl*-K-1; therefore, we begin the assignment from the *ethyl*-K9-*methyl*-K-1 spectrum.^32^ We make the assumption that alkylation has no other effect in the high-frequency range than removal of the corresponding NH stretches. The graphical comparison is shown in Figure 3, and the analysis is reported in Table 1.

**Figure 3:**
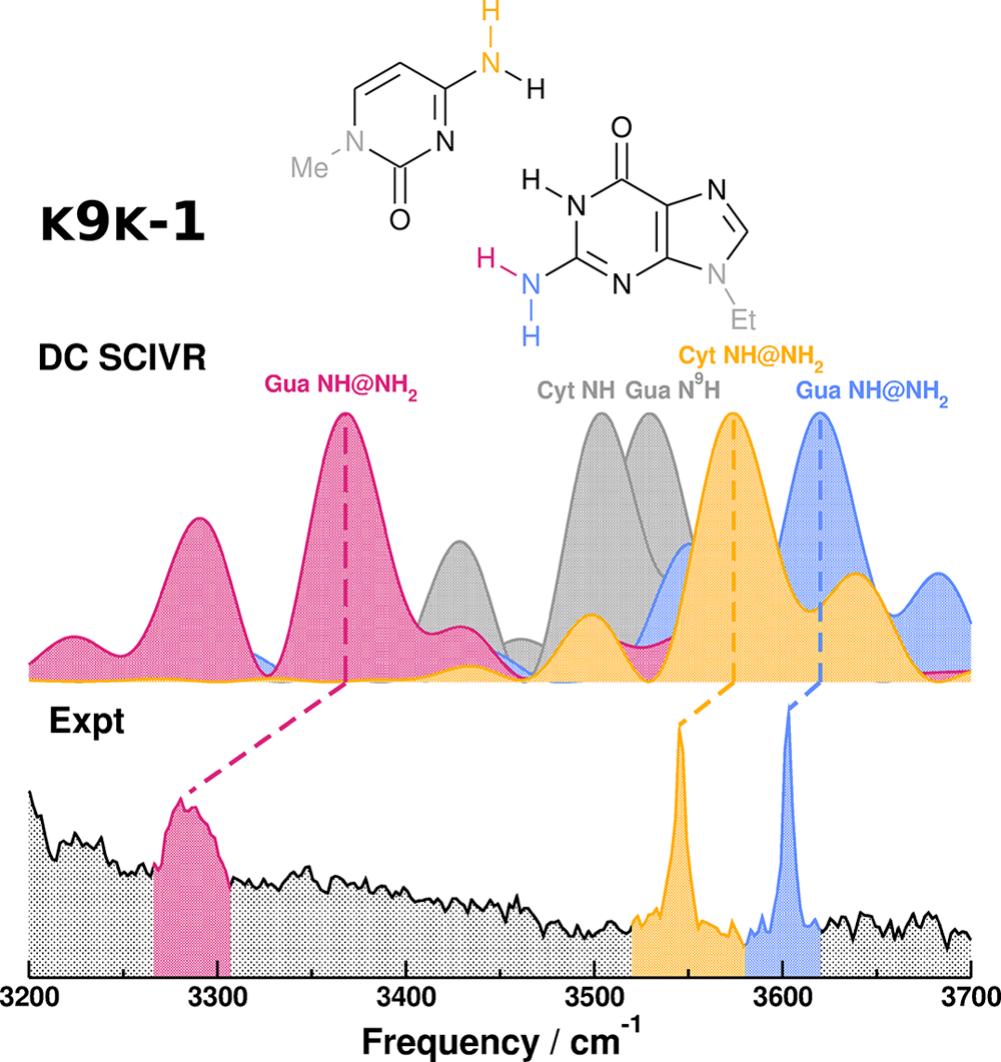
Comparison between the experimental spectra of ethyl-K9-methyl-K-1 and the DC-SCIVR spectra of K9K-1, obtained from a single 25 000 au trajectory, starting from harmonic conditions at the DFT-D/B3LYP def2-TZVP level of theory. The gray peaks are the NH stretches suppressed by the experimental alkylation. The experimental spectrum is reproduced with permission from ref (29). Copyright 2004 National Academy of Sciences.

**Table 1:** Comparison between the DC SCIVR Vibrational Frequencies and the Experimental Frequencies (cm^–1^) for the Main Guanine–Cytosine Pair Tautomers

mode	DC SCIVR^a^	scaled harmonic^b^	experimental^c^
K9K-1			
Gua NH str in NH_2_	3620	3538	3603
Cyt NH str in NH_2_	3574	3526	3545
Gua N^9^H str	3529	3505	alkylated
Cyt NH str	3504	3476	alkylated
Gua NH str in NH_2_	3368	3343	3283
MAE^d^	43	48	
K7E-1			
Cyt OH str	3656	3595	3615
Gua NH_2_ a.str	3534	3547	3520
Cyt NH_2_ a.str	3559	3531	3561
Gua N^7^H str	3536	3511	3543
Gua NH_2_ s.str	3448	3419	3436
MAE^e^	15	25	

a The DC-SCIVR frequencies are obtained from a 25 000 au classical trajectory at the DFT-D/B3LYP def2-TZVP level of theory for each tautomer.

b Scaled harmonic frequencies obtained at the HF/6-31G(d,p) level of theory, using scaling factor 0.893 for NH stretching and 0.867 for OH stretching.^32^

c The experimental frequencies are taken from the ethyl-K9-methyl-K-1 spectrum and the guanine–cytosine pair spectrum (K7E-1).^29^

d Mean absolute error (MAE) is calculated on only three data against experimental results.

e MAE is calculated on five data against experimental results.

All of the spectral features are assigned with good agreement between the experimental and computed frequencies. All of the computed frequencies are blue-shifted in comparison to the experiment. In particular, the guanine NH stretching in NH_2_ (Gua NH@NH_2_) is associated with the broad signal around 3280 cm^–1^. The broad signal is an effect of the strong hydrogen bond, while the larger disagreement between the experimental and DC-SCIVR frequencies of this vibrational mode (in comparison to the other modes) is probably due to the chosen DFT-D/B3LYP level of theory. Indeed, this vibrational frequency is very similar for the harmonic approximation and QCT and DC SCIVR estimates, even for different basis sets (see Table S5 of the Supporting Information). This is quite an unusual behavior, and it suggests that either there is an improper description of the potential energy surface or our assumption breaks down and the experimental alkylation makes a difference for this mode. Nonetheless, we are able to assign this feature because this is the only signal compatible with the guanine NH stretch in the NH_2_ DC-SCIVR frequency. In conclusion, we can exclude the presence of the E9I-1-like tautomer because there is no experimental signal that we can compare to the E9I-1 guanine NH_2_ stretches. This means that we find no spectral evidence of a double proton hopping mechanism.

We then proceed to the assignment of the experimental spectrum obtained by laser desorption of guanine and cytosine, starting with the K7E-1 DC-SCIVR spectra, as suggested in the literature.^29,32,34^ The graphical comparison is shown in Figure 4, and the analysis is reported in Table 1. Our assignment of the experimental signals differs from the assignment previously reported by de Vries et al., and the main reason is that DC SCIVR fully accounts for mode anharmonicity, while a rough scaled harmonic approximation has been previously employed by those same authors for their assignment. Specifically, the modes responsible for the three-pronged structure of the experimental spectrum are in a different order from the previous experimental assignment: the peak at 3561 cm^–1^ is assigned to cytosine NH_2_ stretching instead of guanine NH_2_ asymmetric stretching; the peak at 3543 cm^–1^ is assigned to guanine N^7^H stretching instead of cytosine NH_2_ stretching; and the peak at 3520 cm^–1^ is assigned to guanine NH_2_ asymmetric stretching instead of guanine N^7^H stretching. Our main argument to assign the experimental spectrum to the K7E-1 tautomer and rule out the K9K-1 tautomer is that the experimental spectrum lacks the broad signal at around 3280 cm^–1^, which is present in the ethyl-K9-methyl-K-1 spectrum as a result of the K9K-1 guanine NH stretch in NH_2_, a mode heavily influenced by the hydrogen bond with the cytosine ketonic function. The experimental spectrum shown in Figure 4 is instead characterized by a sharp peak at 3436 cm^–1^, which is compatible with the K7E-1 guanine symmetric NH_2_ stretch. We can also exclude the presence in the experiment of the E9I-1 tautomer, first of all, because E9I-1 would be obtained by means of double proton hopping from the more stable K9K-1 tautomer, and if the latter is missing, it is unlikely that the former is present. Then, because the highest frequency mode of E9I-1, the cytosine NH stretch at 3552 cm^–1^ is too low in frequency to match the experimental signal at 3615 cm^–1^. This is, despite our QCT and DC SCIVR frequencies being even blue-shifted in comparison to the experiment, presumably due to the approximate description of hydrogen bonds and other interactions at the chosen affordable level of electronic theory. The presence of K9E-1 remains to be ruled out, which is lower in energy than K7E-1 and whose high-frequency spectrum is basically indistinguishable from that of K7E-1. To this end, we performed two QCT simulations (one per tautomer) of the out-of-plane NH bending because de Vries and co-workers could not assign any signal above 500 cm^–1^ in their experiments to this vibrational mode. The scaled harmonic calculations suggested for the out-of-plane NH bending a frequency of 477 cm^–1^ for the K7E-1 tautomer and a frequency of 508 cm^–1^ for the K9E-1 tautomer.^34^ The missing peak just above 500 cm^–1^ allowed de Vries and co-workers to rule out the presence of K9E-1 in the experimental spectra. Our QCT simulations confirm and strengthen this conclusion, estimating the target bending at 426 cm^–1^ for K7E-1 and 509 cm^–1^ for K9E-1. Therefore, following the reasoning by de Vries and co-workers, we also rule out the presence of K9E-1 in the experimental spectra. A figure reporting the outcome of these QCT calculations can be found in Figure S1 of the Supporting Information.

**Figure 4:**
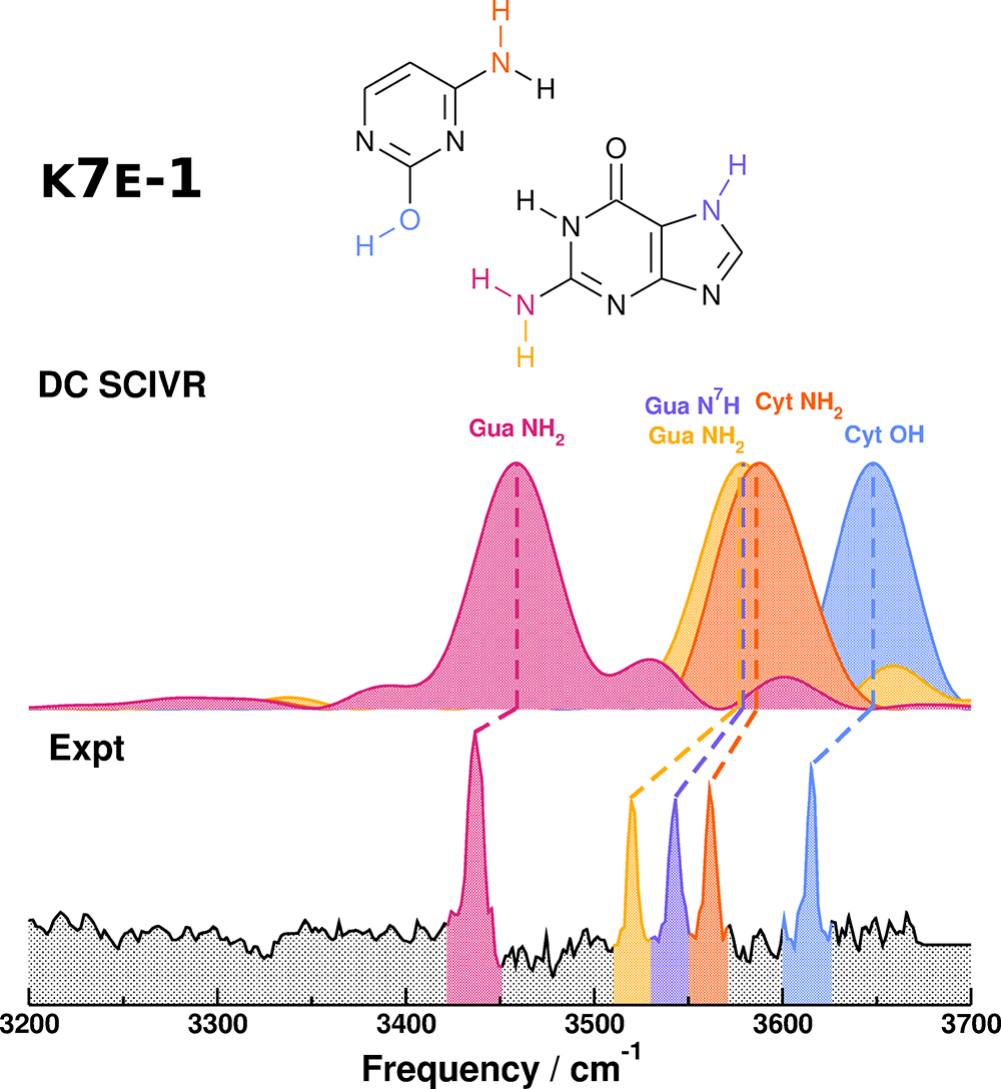
Comparison between the experimental spectra of the guanine–cytosine dimer and the DC-SCIVR spectra of K7E-1, obtained from a single 25 000 au trajectory, starting from harmonic conditions at the DFT-D/B3LYP def2-TZVP level of theory. The experimental spectrum is reproduced with permission from ref (29). Copyright 2004 National Academy of Sciences U.S.A..

Using DC-SCIVR and QCT simulations, we obtained the vibrational power spectra of two tautomers of the guanine–cytosine pair. By comparison of our results to the experimental spectra found in the literature,^29,32^ we managed to assign the relevant spectral features in the high-frequency region of two experimental spectra: one for the isolated guanine–cytosine dimer and the other for its alkylated form. Indeed, the presence of a peak at 3436 cm^–1^ in the spectrum of the guanine–cytosine dimer is the signature feature of a high-energy keto–enol tautomer (K7E-1), because the NH peak in the experimental spectrum of the alkylated Watson-and-Crick tautomer (K9K-1) is at a lower frequency. This peak corresponds to the vibration of the guanine NH_2_ group, which points toward either the ketonic (for K9K-1) or enolic (for K7E-1) form of cytosine. In K7E-1, the peak has been anticipated at higher frequencies (3448 cm^–1^), with a longer O···HN distance (2.05 Å), whereas K9K-1 shows the corresponding peak at lower frequencies (3368 cm^–1^) and a shorter O···HN distance (1.89 Å). In terms of normal modes, K7E-1 is characterized by the usual symmetric/asymmetric motions for NH_2_ stretching. Conversely, in K9K-1 the two NH atoms of guanine NH_2_ are uncoupled and vibrate separately. This means that, in K7E-1, the hydrogen bond is weaker than the hydrogen bond in K9K-1, resulting in a smaller perturbation of the original guanine-free NH_2_ vibrational motion. The fact that the corresponding E9I-1 mode has a QCT frequency of 3435 cm^–1^ and an O···HN distance of 1.94 Å clarifies that the frequency of the guanine NH stretch does depend upon not only the hydrogen bond acceptor but also the relative position of the two bases. Sometimes QCT and DC SCIVR spectra present several peaks (see, for instance, Figure 2). While it is evident which signal corresponds to the target one, side peaks may be due to modes coupled to the target one, in the case of QCT simulations, or to additional combination bands/overtones not detected by means of QCT in the case of the DC-SCIVR calculations.

Our assignment agrees with the literature,^29,32,33,37^ where the spectrum of Figure 4 is assigned to the higher energy keto–enol form (K7E-1) and there is no fingerprint of the global minimum Watson-and-Crick form (WC or K9K-1). The WC form distinctive features appear in the experimental spectrum only when the keto–enol tautomerization is prevented by alkylation.^29^ The absence of the WC form is probably due to the short lifetime of its excited state in the UV pump phase.^29,32^ Theoretical calculations by Domcke et al. suggest that an internal conversion co-adiuvated by the crossing with two doorway states may occur.^38^

It must be noted that in the high-frequency region, we cannot distinguish between K7E-1 and K9E-1, given that their QCT signals lie well within the peak width. However, this does not jeopardize our conclusions because K7E-1 and K9E-1 only differ for the position of a hydrogen not involved in the hydrogen bonds. Furthermore, an examination of the low-frequency region allowed us to differentiate between the two tautomers and to rule out the presence of K9E-1 in the spectrum.

Eventually, we can rule out the presence of the tautomeric form (E9I-1) originated via double proton hopping from the Watson-and-Crick form (WC or K9K-1). Indeed, distinctive signals of this enol tautomer are absent in the experiments. Specifically, the highest frequency transition, attributed to the cytosine-free NH stretching, is at too low-frequency values, and the guanine NH_2_ asymmetric stretching is absent. This suggests that no hopping mechanism is detectable for the nucleobase pair under the IR–UV hole burning experimental conditions. In the past, we demonstrated that NH_2_ rotors can be characterized by quantum spectroscopic features, which can be detected by DC-SCIVR simulations but not by QCT simulations.^25^ This aspect together with the hypothesis that a quantum effect like tunneling could play a major role in the interconversion of the guanine–cytosine tautomers led us to perform calculations employing both DC SCIVR and QCT. The result is that there are no clear differences in the spectra obtained with the two approaches, with equivalent fundamental frequencies of vibrations strengthening our conclusions. Furthermore, in our calculations for the power spectra of E9I-1, we find that peaks are characterized by a much larger full width at half maximum (about 125 cm^–1^ against about 50 cm^–1^ for the other tautomers). This is evidence that E9I-1 is a metastable state and is about to convert to the WC tautomer despite the very short time of the simulation (about 600 fs). This is in agreement with recent calculations of the kinetic constant for the conversion from E9I-1 to K9K-1 by Angiolari et al.^64^ Those calculations illustrate that this process is very fast and influenced by the environment. If the mutagenesis of base coupling is mediated by the double proton hopping mechanism, it means that it happens in a very short time, much faster than conversion to the WC form. We notice that quantum effects are usually more evident in the gas phase than for solvated systems; therefore, our investigation hints at the possibility that quantum effects do not play a major role for DNA in solution. More investigations are indeed necessary on this point. For a better characterization of the mechanism, future spectroscopic investigations (both at experimental and theoretical levels) should include the role of the environment. For instance, going beyond optical spectroscopy, this is being done relative to nuclear magnetic resonance (NMR) experiments. Recent calculations by Slocombe, Sacchi, and co-workers^9^ on tunneling rates in the guanine–thymine pair are compatible with NMR rates, opening the possibility that quantum tunneling plays an effective role in DNA transcription errors.

## Supporting Information Available

(1) Low-frequency analysis for K7E-1 and K9E-1, (2) tautomer energies, summary of the experimental assignment, comparison of the harmonic frequencies, comparison between harmonic and anharmonic (QCT) frequencies, and QCT and DC SCIVR frequencies for K9K-1 NH stretching in NH_2_, and (3) further information on the semiclassical methods employed (PDF)
Transparent Peer Review report available (PDF)

## References

[ref1] Kim S. K.; Takahashi M.; Nordén B. Binding of RecA to Anti-Parallel Poly(dA)·2poly(dT) Triple Helix DNA. Biochim. Biophys. Acta, Gene Struct. Expression 1995, 1264, 129–133. 10.1016/0167-4781(95)00137-6. 7578246

[ref2] Hoogsteen K. The Crystal and Molecular Structure of a Hydrogen-Bonded Complex between 1-Methylthymine and 9-Methyladenine. Acta Crystallogr. 1963, 16, 907–916. 10.1107/S0365110X63002437.

[ref3] Moscato D.; Gabas F.; Conte R.; Ceotto M. Vibrational Spectroscopy Simulation of Solvation Effects on a G-quadruplex. J. Biomol. Struct. Dyn. 2023, 1–11. 10.1080/07391102.2023.2180435. 36856120

[ref4] Watson J. D.; Crick F. H. C. The Structure of DNA. Cold Spring Harb. Symp. Quant. Biol. 1953, 18, 123–131. 10.1101/SQB.1953.018.01.020. 13168976

[ref5] Lemieux S. RNA Canonical and Non-Canonical Base Pairing Types: A Recognition Method and Complete Repertoire. Nucleic Acids Res. 2002, 30, 4250–4263. 10.1093/nar/gkf540. 12364604 PMC140540

[ref6] Löwdin P.-O. Proton Tunneling in DNA and Its Biological Implications. Rev. Mod. Phys. 1963, 35, 724–732. 10.1103/RevModPhys.35.724.

[ref7] Slocombe L.; Al-Khalili J. S.; Sacchi M. Quantum and Classical Effects in DNA Point Mutations: Watson–Crick Tautomerism in AT and GC Base Pairs. Phys. Chem. Chem. Phys. 2021, 23, 4141–4150. 10.1039/D0CP05781A. 33533770

[ref8] Slocombe L.; Sacchi M.; Al-Khalili J. An Open Quantum Systems Approach to Proton Tunnelling in DNA. Commun. Phys. 2022, 5, 109 10.1038/s42005-022-00881-8.

[ref9] Slocombe L.; Winokan M.; Al-Khalili J.; Sacchi M. Quantum Tunnelling Effects in the Guanine-Thymine Wobble Misincorporation via Tautomerism. J. Phys. Chem. Lett. 2023, 14, 9–15. 10.1021/acs.jpclett.2c03171. 36562711 PMC9841559

[ref10] Soler-Polo D.; Mendieta-Moreno J. I.; Trabada D. G.; Mendieta J.; Ortega J. Proton Transfer in Guanine-Cytosine Base Pairs in B-DNA. J. Chem. Theory Comput. 2019, 15, 6984–6991. 10.1021/acs.jctc.9b00757. 31665604

[ref11] Umesaki K.; Odai K. A Kinetic Approach to Double Proton Transfer in Watson–Crick DNA Base Pairs. J. Phys. Chem. B 2020, 124, 1715–1722. 10.1021/acs.jpcb.9b11874. 32045241

[ref12] Gheorghiu A.; Coveney P. V.; Arabi A. A. The Influence of Base Pair Tautomerism on Single Point Mutations in Aqueous DNA. Interface Focus 2020, 10, 20190120 10.1098/rsfs.2019.0120. 33178413 PMC7653342

[ref13] Hartweg S.; Hochlaf M.; Garcia G. A.; Nahon L. Photoionization Dynamics and Proton Transfer within the Adenine-Thymine Nucleobase Pair. J. Phys. Chem. Lett. 2023, 14, 3698–3705. 10.1021/acs.jpclett.3c00564. 37040591

[ref14] Rangadurai A.; Szymanski E. S.; Kimsey I.; Shi H.; Al-Hashimi H. M. Probing Conformational Transitions towards Mutagenic Watson–Crick-like G·T Mismatches Using off-Resonance Sugar Carbon R1ρ Relaxation Dispersion. J. Biomol NMR 2020, 74, 457–471. 10.1007/s10858-020-00337-7. 32789613 PMC7508749

[ref15] Botti G.; Ceotto M.; Conte R. On-the-Fly Adiabatically Switched Semiclassical Initial Value Representation Molecular Dynamics for Vibrational Spectroscopy of Biomolecules. J. Chem. Phys. 2021, 155, 234102 10.1063/5.0075220. 34937370

[ref16] Botti G.; Aieta C.; Conte R. The Complex Vibrational Spectrum of Proline Explained through the Adiabatically Switched Semiclassical Initial Value Representation. J. Chem. Phys. 2022, 156, 164303 10.1063/5.0089720. 35490010

[ref17] Fischer T. L.; Wagner T.; Gottschalk H. C.; Nejad A.; Suhm M. A. A Rather Universal Vibrational Resonance in 1:1 Hydrates of Carbonyl Compounds. J. Phys. Chem. Lett. 2021, 12, 138–144. 10.1021/acs.jpclett.0c03197. 33315407

[ref18] Kabeláč M.; Ryjáček F.; Hobza P. Already Two Water Molecules Change Planar H-bonded Structures of the Adenine···thymine Base Pair to the Stacked Ones: A Molecular Dynamics Simulations Study. Phys. Chem. Chem. Phys. 2000, 2, 4906–4909. 10.1039/b007167f.

[ref19] Seki T.; Chiang K.-Y.; Yu C.-C.; Yu X.; Okuno M.; Hunger J.; Nagata Y.; Bonn M. The Bending Mode of Water: A Powerful Probe for Hydrogen Bond Structure of Aqueous Systems. J. Phys. Chem. Lett. 2020, 11, 8459–8469. 10.1021/acs.jpclett.0c01259. 32931284 PMC7584361

[ref20] Fecko C. J.; Eaves J. D.; Loparo J. J.; Tokmakoff A.; Geissler P. L. Ultrafast Hydrogen-Bond Dynamics in the Infrared Spectroscopy of Water. Science 2003, 301, 1698–1702. 10.1126/science.1087251. 14500975

[ref21] Dean J. L. S.; Fournier J. A. Vibrational Dynamics of the Intramolecular H-Bond in Acetylacetone Investigated with Transient and 2D IR Spectroscopy. J. Phys. Chem. B 2022, 126, 3551–3562. 10.1021/acs.jpcb.2c00793. 35536173

[ref22] Biswal H. S.; Gloaguen E.; Loquais Y.; Tardivel B.; Mons M. Strength of NH···S Hydrogen Bonds in Methionine Residues Revealed by Gas-Phase IR/UV Spectroscopy. J. Phys. Chem. Lett. 2012, 3, 755–759. 10.1021/jz300207k. 26286285

[ref23] Morawietz T.; Urbina A. S.; Wise P. K.; Wu X.; Lu W.; Ben-Amotz D.; Markland T. E. Hiding in the Crowd: Spectral Signatures of Overcoordinated Hydrogen-Bond Environments. J. Phys. Chem. Lett. 2019, 10, 6067–6073. 10.1021/acs.jpclett.9b01781. 31549833

[ref24] Gabas F.; Conte R.; Ceotto M. Quantum Vibrational Spectroscopy of Explicitly Solvated Thymidine in Semiclassical Approximation. J. Phys. Chem. Lett. 2022, 13, 1350–1355. 10.1021/acs.jpclett.1c04087. 35109652 PMC8842300

[ref25] Gabas F.; Di Liberto G.; Conte R.; Ceotto M. Protonated Glycine Supramolecular Systems: The Need for Quantum Dynamics. Chem. Sci. 2018, 9, 7894–7901. 10.1039/C8SC03041C. 30542548 PMC6237109

[ref26] Bertaina G.; Di Liberto G.; Ceotto M. Reduced Rovibrational Coupling Cartesian Dynamics for Semiclassical Calculations: Application to the Spectrum of the Zundel Cation. J. Chem. Phys. 2019, 151, 114307 10.1063/1.5114616. 31542046

[ref27] Fallacara E.; Depondt P.; Huppert S.; Ceotto M.; Finocchi F. Thermal and Nuclear Quantum Effects at the Antiferroelectric to Paraelectric Phase Transition in KOH and KOD Crystals. J. Phys. Chem. C 2021, 125, 22328–22334. 10.1021/acs.jpcc.1c06953. PMC8782431 35082961

[ref28] Fischer T. L.; Bodecker M.; Schweer S. M.; Dupont J.; Lepère V.; Zehnacker-Rentien A.; Suhm M. A.; Schröder B.; Henkes T.; Andrada D. M.; Balabin R. M.; Singh H. K.; Bhattacharyya H. P.; Sarma M.; Käser S.; Töpfer K.; Vazquez-Salazar L. I.; Boittier E. D.; Meuwly M.; Mandelli G.; Lanzi C.; Conte R.; Ceotto M.; Dietrich F.; Cisternas V.; Gnanasekaran R.; Hippler M.; Jarraya M.; Hochlaf M.; Viswanathan N.; Nevolianis T.; Rath G.; Kopp W. A.; Leonhard K.; Mata R. A. The First HyDRA Challenge for Computational Vibrational Spectroscopy. Phys. Chem. Chem. Phys. 2023, 25, 22089–22102. 10.1039/D3CP01216F. 37610422

[ref29] Abo-Riziq A.; Grace L.; Nir E.; Kabeláč M.; Hobza P.; de Vries M. S. Photochemical Selectivity in Guanine – Cytosine Base-Pair Structures. Proc. Natl. Acad. Sci. U. S. A. 2005, 102, 20–23. 10.1073/pnas.0408574102. 15618394 PMC544067

[ref30] Kabeláč M.; Hobza P. At Nonzero Temperatures, Stacked Structures of Methylated Nucleic Acid Base Pairs and Microhydrated Nonmethylated Nucleic Acid Base Pairs Are Favored over Planar Hydrogen-Bonded Structures: A Molecular Dynamics Simulations Study. Chem. - Eur. J. 2001, 7, 2067–2074. 10.1002/1521-3765(20010518)7:10<2067::AID-CHEM2067>3.0.CO;2-S. 11411979

[ref31] Nir E.; Janzen C.; Imhof P.; Kleinermanns K.; de Vries M. S. Guanine Tautomerism Revealed by UV–UV and IR–UV Hole Burning Spectroscopy. J. Chem. Phys. 2001, 115, 4604–4611. 10.1063/1.1391443.

[ref32] Nir E.; Janzen C.; Imhof P.; Kleinermanns K.; de Vries M. S. Pairing of the Nucleobases Guanine and Cytosine in the Gas Phase Studied by IR–UV Double-Resonance Spectroscopy and Ab Initio Calculations. Phys. Chem. Chem. Phys. 2002, 4, 732–739. 10.1039/b107429f.

[ref33] Nir E.; Plützer C.; Kleinermanns K.; de Vries M. Properties of Isolated DNA Bases, Base Pairs and Nucleosides Examined by Laser Spectroscopy. Eur. Phys. J. D 2002, 20, 317–329. 10.1140/epjd/e2002-00167-2.

[ref34] Bakker J. M.; Compagnon I.; Meijer G.; von Helden G.; Kabeláč M.; Hobza P.; de Vries M. S. The Mid-IR Absorption Spectrum of Gas-Phase Clusters of the Nucleobases Guanine and Cytosine. Phys. Chem. Chem. Phys. 2004, 6, 2810–2815. 10.1039/B316158G.

[ref35] Plützer C.; Hünig I.; Kleinermanns K.; Nir E.; de Vries M. S. Pairing of Isolated Nucleobases: Double Resonance Laser Spectroscopy of Adenine-Thymine. ChemPhysChem 2003, 4, 838–842. 10.1002/cphc.200300648. 12961981

[ref36] Plützer C.; Hünig I.; Kleinermanns K. Pairing of the Nucleobase Adenine Studied by IR-UV Double-Resonance Spectroscopy and Ab Initio Calculations. Phys. Chem. Chem. Phys. 2003, 5, 1158–1163. 10.1039/b212338j.

[ref37] Brauer B.; Gerber R. B.; Kabeláč M.; Hobza P.; Bakker J. M.; Abo Riziq A. G.; de Vries M. S. Vibrational Spectroscopy of the G···C Base Pair: Experiment, Harmonic and Anharmonic Calculations, and the Nature of the Anharmonic Couplings. J. Phys. Chem. A 2005, 109, 6974–6984. 10.1021/jp051767m. 16834057

[ref38] Sobolewski A. L.; Domcke W. Ab Initio Studies on the Photophysics of the Guanine– Cytosine Base Pair. Phys. Chem. Chem. Phys. 2004, 6, 2763–2771. 10.1039/B314419D.

[ref39] Jiang Y.; Wang L. Development of Vibrational Frequency Maps for Nucleobases. J. Phys. Chem. B 2019, 123, 5791–5804. 10.1021/acs.jpcb.9b04633. 31260308 PMC6820520

[ref40] Fornaro T.; Biczysko M.; Monti S.; Barone V. Dispersion Corrected DFT Approaches for Anharmonic Vibrational Frequency Calculations: Nucleobases and Their Dimers. Phys. Chem. Chem. Phys. 2014, 16, 10112–10128. 10.1039/C3CP54724H. 24531740 PMC4612423

[ref41] Vaniček J. J. L. Family of Gaussian Wavepacket Dynamics Methods from the Perspective of a Nonlinear Schrödinger Equation. J. Chem. Phys. 2023, 159, 014114 10.1063/5.0146680. 37417753

[ref42] Malpathak S.; Church M. S.; Ananth N. A Semiclassical Framework for Mixed Quantum Classical Dynamics. J. Phys. Chem. A 2022, 126, 6359–6375. 10.1021/acs.jpca.2c03467. 36070472

[ref43] Ceotto M.; Di Liberto G.; Conte R. Semiclassical “Divide-and-Conquer” Method for Spectroscopic Calculations of High Dimensional Molecular Systems. Phys. Rev. Lett. 2017, 119, 010401 10.1103/PhysRevLett.119.010401. 28731742

[ref44] Di Liberto G.; Conte R.; Ceotto M. “Divide and Conquer” Semiclassical Molecular Dynamics: A Practical Method for Spectroscopic Calculations of High Dimensional Molecular Systems. J. Chem. Phys. 2018, 148, 014307 10.1063/1.5010388. 29306274

[ref45] Di Liberto G.; Conte R.; Ceotto M. “Divide-and-conquer” Semiclassical Molecular Dynamics: An Application to Water Clusters. J. Chem. Phys. 2018, 148, 104302 10.1063/1.5023155. 29544263

[ref46] Gandolfi M.; Rognoni A.; Aieta C.; Conte R.; Ceotto M. Machine Learning for Vibrational Spectroscopy via Divide-and-Conquer Semiclassical Initial Value Representation Molecular Dynamics with Application to *N*-Methylacetamide. J. Chem. Phys. 2020, 153, 204104 10.1063/5.0031892. 33261493

[ref47] Gabas F.; Di Liberto G.; Ceotto M. Vibrational Investigation of Nucleobases by Means of Divide and Conquer Semiclassical Dynamics. J. Chem. Phys. 2019, 150, 224107 10.1063/1.5100503. 31202241

[ref48] Gabas F.; Conte R.; Ceotto M. Semiclassical Vibrational Spectroscopy of Biological Molecules Using Force Fields. J. Chem. Theory Comput. 2020, 16, 3476–3485. 10.1021/acs.jctc.0c00127. 32374992 PMC7901649

[ref49] Miller W. H. Semiclassical Theory of Atom–Diatom Collisions: Path Integrals and the Classical S Matrix. J. Chem. Phys. 1970, 53, 1949–1959. 10.1063/1.1674275.

[ref50] Elran Y.; Kay K. G. Improving the Efficiency of the Herman–Kluk Propagator by Time Integration. J. Chem. Phys. 1999, 110, 3653–3659. 10.1063/1.478255.

[ref51] Elran Y.; Kay K. G. Time-Integrated Form of the Semiclassical Initial Value Method. J. Chem. Phys. 1999, 110, 8912–8918. 10.1063/1.478810.

[ref52] Kaledin A. L.; Miller W. H. Time Averaging the Semiclassical Initial Value Representation for the Calculation of Vibrational Energy Levels. II. Application to H_2_CO, NH_3_, CH_4_, CH_2_D_2_. J. Chem. Phys. 2003, 119, 3078–3084. 10.1063/1.1589477.

[ref53] Herman M. F.; Kluk E. A Semiclasical Justification for the Use of Non-Spreading Wavepackets in Dynamics Calculations. Chem. Phys. 1984, 91, 27–34. 10.1016/0301-0104(84)80039-7.

[ref54] Ceotto M.; Atahan S.; Tantardini G. F.; Aspuru-Guzik A. Multiple Coherent States for First-Principles Semiclassical Initial Value Representation Molecular Dynamics. J. Chem. Phys. 2009, 130, 234113 10.1063/1.3155062. 19548717

[ref55] Ceotto M.; Atahan S.; Shim S.; Tantardini G. F.; Aspuru-Guzik A. First-Principles Semiclassical Initial Value Representation Molecular Dynamics. Phys. Chem. Chem. Phys. 2009, 11, 3861 10.1039/b820785b. 19440613

[ref56] Conte R.; Aspuru-Guzik A.; Ceotto M. Reproducing Deep Tunneling Splittings, Resonances, and Quantum Frequencies in Vibrational Spectra From a Handful of Direct Ab Initio Semiclassical Trajectories. J. Phys. Chem. Lett. 2013, 4, 3407–3412. 10.1021/jz401603f. 26705583

[ref57] Gabas F.; Conte R.; Ceotto M. On-the-Fly Ab Initio Semiclassical Calculation of Glycine Vibrational Spectrum. J. Chem. Theory Comput. 2017, 13, 2378–2388. 10.1021/acs.jctc.6b01018. 28489368 PMC5472367

[ref58] De Leon N.; Heller E. J. Semiclassical Quantization and Extraction of Eigenfunctions Using Arbitrary Trajectories. J. Chem. Phys. 1983, 78, 4005–4017. 10.1063/1.445126.

[ref59] Cazzaniga M.; Micciarelli M.; Moriggi F.; Mahmoud A.; Gabas F.; Ceotto M. Anharmonic Calculations of Vibrational Spectra for Molecular Adsorbates: A Divide-and-Conquer Semiclassical Molecular Dynamics Approach. J. Chem. Phys. 2020, 152, 104104 10.1063/1.5142682. 32171221

[ref60] Rognoni A.; Conte R.; Ceotto M. Caldeira–Leggett Model vs *Ab Initio* Potential: A Vibrational Spectroscopy Test of Water Solvation. J. Chem. Phys. 2021, 154, 094106 10.1063/5.0040494. 33685187

[ref61] Rognoni A.; Conte R.; Ceotto M. How Many Water Molecules Are Needed to Solvate One?. Chem. Sci. 2021, 12, 2060–2064. 10.1039/D0SC05785A. PMC8179311 34163968

[ref62] Weigend F.; Ahlrichs R. Balanced Basis Sets of Split Valence, Triple Zeta Valence and Quadruple Zeta Valence Quality for H to Rn: Design and Assessment of Accuracy. Phys. Chem. Chem. Phys. 2005, 7, 3297 10.1039/b508541a. 16240044

[ref63] Grimme S.; Antony J.; Ehrlich S.; Krieg H. A Consistent and Accurate *Ab Initio* Parametrization of Density Functional Dispersion Correction (DFT-D) for the 94 Elements H-Pu. J. Chem. Phys. 2010, 132, 154104 10.1063/1.3382344. 20423165

[ref64] Angiolari F.; Huppert S.; Pietrucci F.; Spezia R. Environmental and Nuclear Quantum Effects on Double Proton Transfer in the Guanine–Cytosine Base Pair. J. Phys. Chem. Lett. 2023, 14, 5102–5108. 10.1021/acs.jpclett.3c00747. 37249365

