# Supplementary material for: Investigating the Spectroscopy of the Gas Phase Guanine–Cytosine Pair: Keto versus Enol Configurations
Source: J Phys Chem Lett. 2023 Sep 28;14(40):8940–7. doi: 10.1021/acs.jpclett.3c02073 (PMC10577776; doi:10.1021/acs.jpclett.3c02073)
Supplement: Supplementary file 1 — jz3c02073_si_001.pdf [file jz3c02073_si_001.pdf]

Supporting Information for “Investigating the Spectroscopy of the Gas Phase Guanine-Cytosine Pair: Keto *vs* Enol Configurations”

Giacomo Botti, Michele Ceotto, and Riccardo Conte*

Dipartimento di Chimica, Università degli Studi di Milano, via Golgi 19, 20133 Milano, Italy

E-mail: riccardo.conte1@unimi.it

Low-frequency region

Table S1: Comparison between the Quasi-Classical Trajectory (QCT) and scaled harmonic frequencies (in cm^{-1}) for the N^7H and N^9H out-of-plane bending of K7E-1 and K9K-1 respectively; scaled harmonic frequencies by Bakker *et al.*[1]

	QCT	Scaled Harmonic
N^7H oop bnd	426	477
N^9H oop bnd	506	515

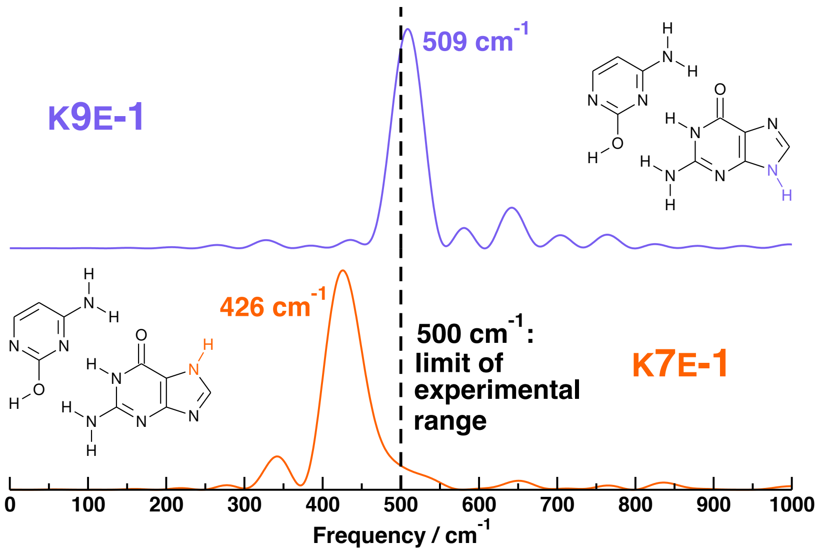

Figure S1: Quasi-Classical Trajectory (QCT) spectra and frequencies for the N^7H (orange) and N^9H (blue) out-of-plane bending of K7E-1 and K9K-1 respectively; the limit of the experimental range is shown as dashed black line.[1]

Tables

Table S2: Comparison between the minimum energies (Hartree) of the four examined tautomers, at theory level DFT-D/B3LYP def2-TZVP. ΔE is the energy gap from K9K-1

Tautomer	K9K-1	K7E-1	E9I-1	K9E-1
E / E_h	-937.9355465	-937.9209256	-937.9193547	-937.9218620
ΔE / E_h	0	0.01462088	0.01619172	0.0136845
ΔE / cm^{-1}	0	3209	3554	3003

Table S3: Experimental assignment of the hole burning IR-UV TOF spectra obtained by de Vries *et al.*, based on the isolated bases vibrational spectra and scaled harmonic frequencies.[1–3] The frequencies are in cm^{-1}.

K7E-1 (expt.)	
Mode	Frequency
Cyt OH str	3615
Gua NH_2 a.str	3561
Cyt NH_2 a.str	3543
Gua N^7H str	3520
Gua NH_2 s.str	3436

Table S4: Comparison between the QCT frequencies (QCT, in cm^{-1}) of the normal modes above 3300 cm^{-1} for the four tautomers, and the harmonic frequencies at theory level DFT-D/B3LYP def2-TZVP (Harm., in cm^{-1}); the QCT frequencies where obtained from a single 25 000 au trajectory at theory level DFT-D/B3LYP def2-TZVP. Double harmonic intensities (Int., in $km\,mol^{-1}$) are included

K9K-1				
Mode	Motion	Harm.	Int.	QCT
81	Gua NH@NH_2 str	3686	117	3621
80	Cyt NH@NH_2 str	3667	86	3574
79	Gua N^9H str	3641	68	3530
78	Cyt NH str	3613	91	3504
77	Gua NH@NH_2 str	3378	1319	3377

E9I-1				
Mode	Motion	Harm.	Int.	QCT
81	Gua NH_2 a.str	3702	136	3550
80	Gua N^9H str	3646	69	3545
79	Cyt NH str	3631	114	3552
78	Cyt im. NH str	3533	17	3441
77	Gua NH_2 s.str	3493	741	3435

K7E-1				
Mode	Motion	Harm.	Int.	QCT
81	Cyt OH str	3757	110	3658
80	Gua NH_2 a.str	3683	163	3533
79	Cyt NH_2 a.str	3675	86	3558
78	Gua N^7H str	3647	84	3533
77	Gua NH_2 s.str	3544	373	3444

K9E-1				
Mode	Motion	Harm.	Int.	QCT
81	Cyt OH str	3757	108	3660
80	Gua NH_2 a.str	3693	161	3549
79	Cyt NH@NH_2 str	3674	85	3581
78	Gua N^9H str	3640	72	3551
77	Gua NH_2 s.str	3539	485	3427

Table S5: Harmonic (Harm), QCT and DC SCIVR frequencies (in cm^{-1}) for K9K-1 guanine NH stretching in NH_2, obtained with 6-31G**, aVDZ and def2-TZVP basis set, using DFT-D/B3LYP level of theory. QCT and DC SCIVR frequencies were obtained with a single 25 000 au trajectory at the indicate level of theory

Basis set	Harm	QCT	DC SCIVR
6-31G**	3389	3374	—
aVDZ	3356	3321	—
def2-TZVP	3378	3377	3368

Semiclassical Approaches

As stated in the letter, the Time-Averaged Semiclassical Initial Value Representation (TA-SCIVR) power spectrum $I(E)$ is able to include quantum effects from multiple classical trajectories. The TA-SCIVR power spectrum is evaluated as

$$I(E) = \left(\frac{1}{2\pi\hbar}\right)^{N_v} \iint d\mathbf{p}_0 d\mathbf{q}_0 \, \frac{1}{2\pi\hbar T} \left| \int_0^T dt \, e^{\frac{i}{\hbar}[S_t(\mathbf{p}_0,\mathbf{q}_0)+Et+\phi_t(\mathbf{p}_0,\mathbf{q}_0)]} \langle\Psi|g_t(\mathbf{p}_0,\mathbf{q}_0)\rangle \right|^2 \quad \text{(S1)}$$

Usually, the time-dependent wavefunction $|g_t\rangle$ is espressed using Heller's coherent states. Coherent states have the following Gaussian representation in the configuration space:[4–8]

$$\langle\mathbf{q}|g_t\rangle = \left(\frac{\det(\Gamma)}{\pi^{N_\nu}}\right)^{1/4} \exp\left\{-(\mathbf{q}-\mathbf{q}_t)^T\frac{\Gamma}{2}(\mathbf{q}-\mathbf{q}_t) + \frac{i}{\hbar}\mathbf{p}_t^T(\mathbf{q}-\mathbf{q}_t)\right\}$$

where Γ is the coherent state width matrix, usually expressed as the diagonal matrix of the harmonic frequencies of vibration.

TA SCIVR uses a time-averaging filter to improve the phase space integration, but still requires thousands of trajectories to compute a single power spectrum. This limits the application of the method to systems that already have a computationally affordable potential energy surface (PES). However, when the system grows in dimensionality, obtaining a reliable PES becomes more and more difficult, and running the trajectory on-the-fly is the most viable option. This means that at each step of the dynamics, the potential energy and forces are evaluated *ab initio*, using an electronic structure software. This greatly reduces the number of trajectories that could be computed at a reasonable computational cost, making the Multiple Coherent Semiclassical Initial Value Representation (MC SCIVR) necessary for *ab initio* potentials.

The reference state usually employed in a MC-SCIVR calculation is of the type[9,10]

$$|\Psi\rangle = \prod_j^{N_v} \epsilon_{1,j}|\mathbf{p}_{\mathrm{eq},j},\mathbf{q}_{\mathrm{eq},j}\rangle + \epsilon_{2,j}|-\mathbf{p}_{\mathrm{eq},j},\mathbf{q}_{\mathrm{eq},j}\rangle$$

where $|(\mathbf{p}_{\text{eq},j}, \mathbf{q}_{\text{eq},j})\rangle$ is a coherent state centered in the equilibrium coordinates and momenta of the j-th vibrational mode. By choosing the parameter $\epsilon_{2,j}$ to be either $+1$ or -1, one can select the symmetry of the reference state, locking in the vibrational signal corresponding to the normal mode of choice and allowing an enhanced sampling of the vibrational signal. Therefore, when employing MC SCIVR, a single tailored trajectory is enough to compute an accurate vibrational power spectrum.[11] The trajectory is usually initialized at the harmonic zero point energy (ZPE) level as the most accessible approximation of the real vibrational energy, but the initial conditions can be improved – at a cost – by running a preparatory trajectory under adiabatic switching conditions.[12] The method is called Adiabatic Switching Semiclassical Initial Value Representation (AS SCIVR) and allows to start the semiclassical trajectory from anharmonic conditions.[13–16]

In Eq.(S1) quantum effects are included mainly in the complex exponential action and in the pre-exponential factor phase. To calculate the pre-exponential factor, *i.e.* the monodromy matrix elements, one needs to calculate the Hessian matrix at each time step of the dynamics. This represents the main bottleneck for MC-SCIVR calculations. The Hessian Database (HDB) method was previously developed by the authors to alleviate this issue.[13] HDB reduces the number of Hessian matrices to be computed by building a database in which these matrices are stored after being computed. Since short vibrational trajectories are periodic, the system keeps visiting the same neighborhood of the phase space. Therefore, when it gets close to a point where the Hessian matrix has been already computed and stored, the stored Hessian is recovered instead of computing the Hessian ex novo. The HDB has recently been improved with the implementation of a neural gas algorithm to efficiently select the points where the Hessian should be computed.[17]

When employing Divide-and-Conquer Semiclassical Initial Value Representation, all elements in Eq.(S1) are straightforwardly projected into a subspace, except for the potential energy V generating the reduced dimensional classical action S. For this part we introduced

an approximate projected potential

$$\tilde{V}(\tilde{\mathbf{q}}_M) = V(\tilde{\mathbf{q}}_M; \mathbf{q}^{\mathrm{eq}}_{N_v-M}) + \lambda$$

where $\tilde{V}$ is the projected potential on the M-dimensional subspace, $V(\tilde{\mathbf{q}}_M; \mathbf{q}^{\mathrm{eq}}_{N_v-M})$ is the potential of the system with all but the M modes fixed at equilibrium position, and λ is an artificial external field of the type

$$\lambda = V(\tilde{\mathbf{q}}_M; \mathbf{q}_{N_v-M}) - \left[V(\tilde{\mathbf{q}}_M; \mathbf{q}^{\mathrm{eq}}_{N_v-M}) + V(\tilde{\mathbf{q}}^{\mathrm{eq}}_M; \mathbf{q}_{N_v-M})\right]$$

The vibrational space is divided into subspaces according to either the Hessian or the Jacobian criteria. The former method groups the normal modes according to the off-diagonal terms of the Hessian matrices,[18,19] while the latter selects the subspaces based on the determinant of the subspace Jacobian matrix closer to unity.[20] This last criterion has been successfully implemented with a machine learning algorithm.[21]

References

(1) Bakker, J. M.; Compagnon, I.; Meijer, G.; von Helden, G.; Kabeláč, M.; Hobza, P.; de Vries, M. S. The Mid-IR Absorption Spectrum of Gas-Phase Clusters of the Nucleobases Guanine and Cytosine. *Phys. Chem. Chem. Phys.* **2004**, *6*, 2810–2815.

(2) Abo-Riziq, A.; Grace, L.; Nir, E.; Kabelac, M.; Hobza, P.; de Vries, M. S. Photochemical Selectivity in Guanine–Cytosine Base-Pair Structures. *Proc. Natl. Acad. Sci.* **2005**, *102*, 20–23.

(3) Nir, E.; Janzen, C.; Imhof, P.; Kleinermanns, K.; de Vries, M. S. Pairing of the Nucleobases Guanine and Cytosine in the Gas Phase Studied by IR–UV Double-Resonance Spectroscopy and Ab Initio Calculations. *Phys. Chem. Chem. Phys.* **2002**, *4*, 732–739.

(4) Herman, M. F.; Kluk, E. A Semiclasical Justification for the Use of Non-Spreading Wavepackets in Dynamics Calculations. *Chem. Phys.* **1984**, *91*, 27–34.

(5) Kay, K. G. Integral Expressions for the Semiclassical Time-dependent Propagator. *J. Chem. Phys.* **1994**, *100*, 4377–4392.

(6) Kay, K. G. Semiclassical Propagation for Multidimensional Systems by an Initial Value Method. *J. Chem. Phys.* **1994**, *101*, 2250–2260.

(7) Kay, K. G. Numerical Study of Semiclassical Initial Value Methods for Dynamics. *J. Chem. Phys.* **1994**, *100*, 4432–4445.

(8) Heller, E. J. Frozen Gaussians: A Very Simple Semiclassical Approximation. *J. Chem. Phys.* **1981**, *75*, 2923–2931.

(9) Ceotto, M.; Atahan, S.; Tantardini, G. F.; Aspuru-Guzik, A. Multiple Coherent States for First-Principles Semiclassical Initial Value Representation Molecular Dynamics. *J. Chem. Phys.* **2009**, *130*, 234113.

(10) Ceotto, M.; Atahan, S.; Shim, S.; Tantardini, G. F.; Aspuru-Guzik, A. First-Principles Semiclassical Initial Value Representation Molecular Dynamics. *Phys. Chem. Chem. Phys.* **2009**, *11*, 3861.

(11) Gabas, F.; Conte, R.; Ceotto, M. On-the-Fly Ab Initio Semiclassical Calculation of Glycine Vibrational Spectrum. *J. Chem. Theory Comput.* **2017**, *13*, 2378–2388.

(12) Landau, L. D.; Lifshitz, E. M. *Mechanics*; Butterworth–Heinemann, Oxford, 1976.

(13) Conte, R.; Gabas, F.; Botti, G.; Zhuang, Y.; Ceotto, M. Semiclassical Vibrational Spectroscopy with Hessian Databases. *J. Chem. Phys.* **2019**, *150*, 244118.

(14) Botti, G.; Ceotto, M.; Conte, R. On-the-Fly Adiabatically Switched Semiclassical Initial Value Representation Molecular Dynamics for Vibrational Spectroscopy of Biomolecules. *J. Chem. Phys.* **2021**, *155*, 234102.

(15) Botti, G.; Aieta, C.; Conte, R. The Complex Vibrational Spectrum of Proline Explained through the Adiabatically Switched Semiclassical Initial Value Representation. *J. Chem. Phys.* **2022**, *156*, 164303.

(16) Conte, R.; Aieta, C.; Botti, G.; Cazzaniga, M.; Gandolfi, M.; Lanzi, C.; Mandelli, G.; Moscato, D.; Ceotto, M. Anharmonicity and Quantum Nuclear Effects in Theoretical Vibrational Spectroscopy: A Molecular Tale of Two Cities. *Theor Chem Acc* **2023**, *142*, 53.

(17) Gandolfi, M.; Ceotto, M. Unsupervised Machine Learning Neural Gas Algorithm for Accurate Evaluations of the Hessian Matrix in Molecular Dynamics. *J. Chem. Theory Comput.* **2021**, *17*, 6733–6746.

(18) Ceotto, M.; Di Liberto, G.; Conte, R. Semiclassical "Divide-and-Conquer" Method for Spectroscopic Calculations of High Dimensional Molecular Systems. *Phys. Rev. Lett.* **2017**, *119*, 010401.

(19) Cazzaniga, M.; Micciarelli, M.; Moriggi, F.; Mahmoud, A.; Gabas, F.; Ceotto, M. Anharmonic Calculations of Vibrational Spectra for Molecular Adsorbates: A Divide-and-Conquer Semiclassical Molecular Dynamics Approach. *J. Chem. Phys.* **2020**, *152*, 104104.

(20) Di Liberto, G.; Conte, R.; Ceotto, M. "Divide and Conquer" Semiclassical Molecular Dynamics: A Practical Method for Spectroscopic Calculations of High Dimensional Molecular Systems. *J. Chem. Phys.* **2018**, *148*, 014307.

(21) Gandolfi, M.; Rognoni, A.; Aieta, C.; Conte, R.; Ceotto, M. Machine Learning for Vibrational Spectroscopy via Divide-and-Conquer Semiclassical Initial Value Representation Molecular Dynamics with Application to N -Methylacetamide. *J. Chem. Phys.* **2020**, *153*, 204104.
