# Supplementary material for: Investigating the Spectroscopy of the Gas Phase Guanine–Cytosine Pair: Keto versus Enol Configurations
Source: J Phys Chem Lett. 2023 Sep 28;14(40):8940–7. doi: 10.1021/acs.jpclett.3c02073 (PMC10577776; doi:10.1021/acs.jpclett.3c02073)
Supplement: Supplementary file 2 — jz3c02073_si_002.pdf [file jz3c02073_si_002.pdf]

jz-2023-02073r.R1

Name: Peer Review Information for "Investigating the Spectroscopy of the Gas Phase Guanine-Cytosine Pair: Keto <i>vs</i> Enol Configurations"

First Round of Reviewer Comments

Reviewer: 1

Comments to the Author

In this article, Botti et al studied the spectroscopy of guanine-cytosine pair by using semi-classical simulations. The dynamical methods are clearly the last and best possible to investigate the spectroscopy of a molecular system and this study is an interesting application of the developments done in the group in last years.

The article is well-written and easy to follow and the topic of interest for the JPCLett readership.

There are few but important point that the authors should elucidate before considering the article publishable. To let them the proper time for revision (and eventually new calculations), I suggest a « major revision ».

1) My main point is that the authors say when discussing results, that this shows that the K7E-1 tautomer agrees with experiments, however I do not clearly see why we should rule out the K9E-1 structure. They evoked it at the end of the article (beginning of page 17 they say « we think that only a thorough examination of the low frequency region could lead to a full differentiation between the two tautomers ». Authors should discuss it also when showing the results.

2) So, in connection to that, they did DC-SCIVR calculations only for K7E-1 and K9K-1 and not for K9E-1. Why ? I imagine that DC-SCIVR is much computationally costly than QCT. However, if I compare Table 1 with Table S2, I can see that QCT provides basically the same results ! This is good, since one can use QCT results to interpret the experiments and assign bands and structure.

3) It is not clear to me the deep comparison done between the methylated experimental band and the calculations (done on the original structure). Surely they occupy a too large place in the article, in particular since at the end few conclusions can be done since there are many aspects which can be at the origin of the discrepancy between theory and experiments: the methods and the system! Authors could go straight to the important point of this part (which is to rule out WC structure, basically).

4) Page 13 they say that the discrepancy with the results by de Vries et al is in the anharmonicity. However, they also used another method. And if I compare harmonic and anharmonic frequencies present in Table S2, anharmonicity provides a red shift with a scaling factor of 0.97-0.98 (as I obtained for some frequencies, I did not go through all of them but …). Of course, in the scaling factors typically used there is also a method correction, since they are obtained by comparing directly with experiments. However, I cannot see that dynamics has a huge impact on frequencies. Authors should be more explicit in comparing results by de Vries reporting their frequencies and mode attribution in the present work.

5) While, of course, the method is reported in previous works, I think that one aspect should be clarified to readers of this article (who are potentially not experts in the specific field): in Eq 2 "pj(t) is the linear momentum of the j-th vibrational normal mode at time t". So the classical trajectory is done in normal modes? Or are they "effective" ? Or calculated on the fly? Or something else? So in Eq 1, p and q are cartesian or normal mode coordinates? Please clarify to let understand how simulations are actually done and what are the key ingredients.

6) It is interesting in spectra reported in Figure 2, that some modes have multiple peaks in the spectra, like e.g. GuaNH@NH2, str in K9K-1. Can they comment on that? Is this a signal that the kind of localization they did (using the pj modes …) is not fully localized and normal modes are in a way delocalized? And then, in this case, which frequencies they report in summarizing tables?

7) In Figure 3, they attributed the experimental peak slightly below 3300 cm-1 to the GuaNH@NH2 band at about 3350 cm-1. But the same mode has a peak which is almost exactly superimposed to the experimental value. Can they comment on that?

Minor points:

- I could be easier for a reader to have sketches of the structures (at least WC and K7E-1) from the beginning since they are discussed in the introduction

- page 8, the say "low frequency region" but for the bending, and then in table S3 they report only high frequency region. This can help in clarifying point (1), but this is never evoked. How the static calculations done at the present level of theory compare them with experiments/calculations reported by de Vries et al?

- Page 14 they say "our QCT and DC-SCIVR frequencies are even blue shifted compared to the experiment". This is what observed, but this is because of the method or because the anharmonicity is not enough? Do they have any clues about the "temperature" of the system in the experiments? If I

understand well, they did IR-UV hole burning spectroscopy after laser desorption, so there can be a lot of energy in the system …

Reviewer: 2

Comments to the Author

In their manuscript, the authors describe a thorough quasi-classical/semi-classical analysis of the vibrational spectra of the guanine-cytosine dimer. Their results confirm the experimental conclusion that the Watson-Crick structure is not detected in the spectra and suggest that double-proton tunneling does not play a role in these spectra. This manuscript goes substantially beyond the previous theoretical assignments based on scaled harmonic models and thus provides a more rigorous theoretical support for previous experimental conclusions.

I have several suggestions:

(1) In the introduction, on line 9 of page 3, it is not clear to me what “at occurrence” means in “hydrogen bond allows the DNA molecule to open and close the double helix at occurrence”.

(2) On page 5, in line 5, it is mentioned that going beyond the scaled-harmonic calculation with a precise quantum assignment would demonstrate the relevance of quantum effects. Could it be simply a better description of anharmonicity (in the quasi-classical and semi-classical methods used) rather than the importance of quantum effects that would explain the improvement over the scaled harmonic results?

(3) In the caption of Fig. 4, it is mentioned that the four selected tautomers are “lowest-energy GC tautomers”, whereas in the main text, it seams to be a more qualitative selection.

(4) Among the main conclusions is the statement on page 13 that the authors can “exclude the presence of the E9I-1 like tautomer” and “find no spectral evidence of a double proton hopping mechanism”. Could the authors explain this in more detail? In Fig. 2, the spectra of K9K-1 and E9I-1 seem quite similar.

(5) Likewise, on page 14, they exclude E9I-1 tautomer. How do they exclude the K9E-1 isomer, which has very a similar spectrum in Fig. 2 to that of K7E-1?

In particular, it would be nice to go slightly deeper into the discussion of points (4) and (5) to support more strongly the main conclusions of a very interesting and otherwise very clearly written manuscript.

Author's Response to Peer Review Comments:

Reply to Referees' Comments

Our reply are in blue. New text in the revised version is in red.

Reviewer 1:

Comments:
In this article, Botti et al studied the spectroscopy of guanine-cytosine pair by using semi-classical simulations. The dynamical methods are clearly the last and best possible to investigate the spectroscopy of a molecular system and this study is an interesting application of the developments done in the group in last years. The article is well-written and easy to follow and the topic of interest for the JPCLett readership. There are few but important point that the authors should elucidate before considering the article publishable. To let them the proper time for revision (and eventually new calculations), I suggest a «major revision».

Authors' reply: We thank the Reviewer for their careful reading of our manuscript and for the overall positive assessment of our paper.

1) My main point is that the authors say when discussing results, that this shows that the K7E-1 tautomer agrees with experiments, however I do not clearly see why we should rule out the K9E-1 structure. They evoked it at the end of the article (beginning of page 17 they say «we think that only a thorough examination of the low frequency region could lead to a full differentiation between the two tautomers». Authors should discuss it also when showing the results.

Authors' reply: We thank the Reviewer for this excellent suggestion. We have now performed two new QCT calculations for the two tautomers (K7E-1 and K9E-1) involving the low frequency region. Just above 500 cm^{-1} (at 514 cm^{-1}), de Vries found in his experiment a single signal with significant intensity (J.M. Bakker, I. Compagnon, G. Meijer, G. von Helden, M. Kabelac, P. Hobza, and M.S. de Vries *Phys. Chem. Chem. Phys.* **6**, 2810 (2004)). This was assigned to a C-O-H in-plane bending. Based on scaled harmonic calculations de Vries found that if the K9E-1 tautomer were present, then a second signal just above 500 cm^{-1}, due to the guanine N-H out-of-plane bending, should be found. The corresponding guanine N-H out-of-plane bending signal for the K7E-1 tautomer was instead anticipated below 500 cm^{-1} meaning that if only the K7E-1 tautomer were present, then a single signal just above 500 cm^{-1} should be found, which is in agreement with the experiment. For this reason, de Vries concluded "we are tempted to assign the spectrum to structure D", D being the K7E-1 tautomer.
Our new QCT simulations confirm and strengthen de Vries' assignment because they anticipate the frequency of the guanine NH out-of-plane bending at 426 cm^{-1} for K7E-1 and at 509 cm^{-1} for K9E-1. Therefore, our conclusion is the same as de Vries and we rule out the presence of the K9E-1 tautomer.
In response to this comment we have inserted a new Figure in the supplementary information file. Furthermore, as suggested by the Reviewer, we have inserted the following new text when discussing the results:
"It remains to rule out the presence of K9E-1, which is lower in energy than K7E-1 and whose high frequency spectrum is basically indistinguishable from that of K7E-1. To this end we performed two QCT simulations (one per tautomer) of the out-of-plane N-H bending because de Vries and co-workers could not assign any signal above 500 cm^{-1} in their experiments to this vibrational mode. The scaled harmonic calculations suggested for the out-of-plane N-H bending a frequency of 477 cm^{-1} for the K7E-1 tautomer, and a frequency of 508 cm^{-1} for the K9E-1 tautomer. The missing peak just above 500 cm^{-1} allowed de Vries and co-workers to rule out the presence of K9E-1 in the experimental spectra. Our QCT simulations confirm and strengthen this conclusion estimating the target bending at 426 cm^{-1} for K7E-1 and at 509 cm^{-1} for K9E-1. Therefore, following de Vries' reasoning, we also rule out the presence of K9E-1 in the experimental spectra. A figure reporting the outcome of QCT calculations can be found in the Supplementary Information (see Figure S1)."
Also, we have modified the text at the beginning of page 17 in the following way:
"Furthermore, an examination of the low frequency region has allowed us to differentiate between the two tautomers and to rule out the presence of K9E-1 in the spectrum".

2) So, in connection to that, they did DC-SCIVR calculations only for K7E-1 and K9K-1 and not for K9E-1. Why? I imagine that DC-SCIVR is much computationally costly than QCT. However, if I compare Table 1 with Table S2, I can see that QCT provides basically the same results! This is good, since one can use QCT results to interpret the experiments and assign bands and structure.

Authors' reply: Generally, QCT and DC-SCIVR simulations are expected to provide different outcomes if quantum effects are relevant, but this does not seem the case for the investigated system at least as far as the focus is on fundamentals. Therefore, in this case we agree with this comment by Reviewer 1 and, as anticipated in the

answer to comment 1), we employed QCT to further validate our conclusion about the absence of the K9E-1 tautomer in the experimental spectrum.

3) It is not clear to me the deep comparison done between the methylated experimental band and the calculations (done on the original structure). Surely they occupy a too large place in the article, in particular since at the end few conclusions can be done since there are many aspects which can be at the origin of the discrepancy between theory and experiments: the methods and the system! Authors could go straight to the important point of this part (which is to rule out WC structure, basically).

Authors' reply: we beg to differ on this point. Ruling out (or, conversely, pointing out) the presence of the WC structure in the experiment is one of the two main goals of our investigation, as clearly stated in the Introduction. We are aware that the experimental system for the WC tautomer is in part different from the simulated one (due to the experimental necessity to perform alkylation to get a spectrum for the WC pair) but, as stated in the paper, we believe the structural differences do not affect significantly the portion of the spectrum we are taking into consideration. For this reason no changes have been made to the original manuscript following this comment.

4) Page 13 they say that the discrepancy with the results by de Vries et al is in the anharmonicity. However, they also used another method. And if I compare harmonic and anharmonic frequencies present in Table S2, anharmonicity provides a red shift with a scaling factor of 0.97-0.98 (as I obtained for some frequencies, I did not go through all of them but …). Of course, in the scaling factors typically used there is also a method correction, since they are obtained by comparing directly with experiments. However, I cannot see that dynamics has a huge impact on frequencies. Authors should be more explicit in comparing results by de Vries reporting their frequencies and mode attribution in the present work.

Authors' reply: We have now added in Table 1 the scaled harmonic frequencies determined by de Vries.

5) While, of course, the method is reported in previous works, I think that one aspect should be clarified to readers of this article (who are potentially not experts in the specific field): in Eq 2 "pj(t) is the linear momentum of the j-th vibrational normal mode at time t". So the classical trajectory is done in normal modes? Or are they "effective" ? Or calculated on the fly? Or something else? So in Eq 1, p and q are cartesian or normal mode coordinates? Please clarify to let understand how simulations are actually done and what are the key ingredients.

Authors' reply: we thank the Reviewer for this comment. p and q are normal mode coordinates, but the dynamics is performed in Cartesian coordinates. The conversion from Cartesian to normal mode coordinates is, as usual, performed by means of the matrix of vibrational eigenvectors. To clarify this point we have added the following text:
"We perform *ab initio* "on-the-fly" Cartesian evolution of the dynamics with Cartesian coordinates and momenta transformed into normal mode coordinates and momenta at each time step along the trajectory."

6) It is interesting in spectra reported in Figure 2, that some modes have multiple peaks in the spectra, like e.g. GuaNH@NH2, str in K9K-1. Can they comment on that? Is this a signal that the kind of localization they did (using the pj modes …) is not fully localized and normal modes are in a way delocalized? And then, in this case, which frequencies they report in summarizing tables?

Authors' reply: if the Reviewer's comment refers to the QCT spectra (colored in Figure 2), then the additional peaks are due to modes coupled with the target one. If she/he refers to the DC-SCIVR spectra (black solid line in Figure 2), then additional peaks are either the same signals detected by QCT but with a different magnitude, or they are due to combination bands/overtones not detected by the QCT calculation. In the tables we adopt the same labels as in the figures for the several modes, so it should be clear which frequencies we report in the tables. Upon this comment, we have added the following short paragraph to our conclusions:
"Sometimes QCT and DC-SCIVR spectra present several peaks (see, for instance, Figure 2). While it is evident which signal correspond to the target one, side peaks may be due to modes coupled to the target one, in the case of QCT simulations, or to additional combination bands/overtones not detected by means of QCT in the case of the DC-SCIVR calculations."

7) In Figure 3, they attributed the experimental peak slightly below 3300 cm-1 to the GuaNH@NH2 band at about 3350 cm-1. But the same mode has a peak which is almost exactly superimposed to the experimental value. Can they comment on that?

Authors' reply: There are multiple reasons for our assignment at around 3350 cm^{-1} instead of around 3300 cm^{-1}. First of all the assigned peak is the most intense one in the relevant QCT spectrum. Secondly, there is no other possible vibrational mode expected at about 3350 cm^{-1}, so the signal at 3350 cm^{-1} would be hard to assign otherwise. Finally we found blue shifts due to the chosen affordable level of electronic theory for the other investigated fundamentals and it is not surprising to get a blue shifted estimate also for this peak. For these reasons, we opted for a rigorous assignment at about 3350cm^{-1}, even if another signal closer to the experiment is present in our calculation.
No changes were made to the manuscript based on this comment.

Minor points:

- I could be easier for a reader to have sketches of the structures (at least WC and K7E-1) from the beginning since they are discussed in the introduction

Authors' reply: The TOC graphic will appear close to the abstract and it already contains the structures of 3 tautomers (WC, K7E-1, E9I-1), which are very helpful for the reader not used to them. So, we prefer to keep Figure 1 in the original place.

- page 8, the say "low frequency region" but for the bending, and then in table S3 they report only high frequency region. This can help in clarifying point (1), but this is never evoked. How the static calculations done at the present level of theory compare them with experiments/calculations reported by de Vries et al?

Authors' reply: we have now added Table S1 in the supporting information file to clarify this point.

- Page 14 they say "our QCT and DC-SCIVR frequencies are even blue shifted compared to the experiment". This is what observed, but this is because of the method or because the anharmonicity is not enough? Do they have any clues about the "temperature" of the system in the experiments? If I understand well, they did IR-UV hole burning spectroscopy after laser desorption, so there can be a lot of energy in the system …

Authors' reply: QCT and DC-SCIVR are able to describe entirely the anharmonicity of the system with excellent accuracy, so we believe that there might be some underestimation of the anharmonicity due to the level of electronic energy employed. We are not aware of the temperature of experiments. Quantum frequencies of vibrations do not depend on temperature, while intensities and band shapes do. However, even if the experimental system is at very high temperature or contains a lot of energy we do not think it is enough to enhance bands far away from the target frequency.
As a consequence of this comment, at the beginning of page 14 we have inserted the following, new text:
",presumably due to the approximate description of hydrogen bonds and other interactions at the chosen affordable level of electronic theory."

Reviewer 2:

Comments:
In their manuscript, the authors describe a thorough quasi-classical/semi-classical analysis of the vibrational spectra of the guanine-cytosine dimer. Their results confirm the experimental conclusion that the Watson-Crick structure is not detected in the spectra and suggest that double-proton tunneling does not play a role in these spectra. This manuscript goes substantially beyond the previous theoretical assignments based on scaled harmonic models and thus provides a more rigorous theoretical support for previous experimental conclusions.

Authors' reply: We thank the Reviewer for his/her careful reading of our manuscript and for the overall positive assessment of the paper.

I have several suggestions:

(1) In the introduction, on line 9 of page 3, it is not clear to me what "at occurrence" means in "hydrogen bond allows the DNA molecule to open and close the double helix at occurrence".

Authors' reply: we have canceled "at occurrence" in our revised manuscript. The text should be now clearer.

(2) On page 5, in line 5, it is mentioned that going beyond the scaled-harmonic calculation with a precise quantum assignment would demonstrate the relevance of quantum effects. Could it be simply a better description of anharmonicity (in the quasi-classical and semi-classical methods used) rather than the importance of quantum effects that would explain the improvement over the scaled harmonic results?

Authors' reply: The Reviewer is perfectly right, and that is actually our same conclusion. We could improve over the scaled harmonic results because our methods are able to fully account for anharmonicity. However, we did not know this a priori and we had to determine whether sizable quantum effects were present or not. To this end, as anticipated in the Introduction, we employed two methods (QCT and DC SCIVR) both able to account for anharmonicity but only one (DC SCIVR) able to get quantum effects. Since the two methods return results in almost perfect agreement (as pointed out also in a comment by Reviewer 1), our conclusion is that it is the anharmonicity that plays the main role and quantum effects are not detectable and/or relevant for the spectra.
No changes to the manuscript have been introduced following this comment.

(3) In the caption of Fig. 4, it is mentioned that the four selected tautomers are "lowest-energy GC tautomers", whereas in the main text, it seams to be a more qualitative selection.

Authors' reply: Did the Referee mean Figure 1? Anyway we thank the Reviewer for this suggestion. Now we have modified the caption of Figure 1 in the following way: "… of the four investigated G-C tautomers …".

(4) Among the main conclusions is the statement on page 13 that the authors can "exclude the presence of the E9I-1 like tautomer" and "find no spectral evidence of a double proton hopping mechanism". Could the authors explain this in more detail? In Fig. 2, the spectra of K9K-1 and E9I-1 seem quite similar.

Authors' reply: We thank the Reviewer for this comment. To address it, we have partially modified and integrated the text at the bottom of page 13 and beginning of page 14 in the following way:
"Our main argument to assign the experimental spectrum to the K7E-1 tautomer and rule out the K9K-1 one is that the experimental spectrum lacks the broad signal at around 3280 cm^{-1}, which is present in the ethyl-K9-methyl-K-1 spectrum due to the K9K-1 guanine NH stretch in NH_2, a mode heavily influenced by the hydrogen bond with the cytosine ketonic function. The experimental spectrum shown in Fig. 4 is instead characterized by a sharp peak at 3436 cm^{-1}, compatible with the K7E-1 guanine symmetric NH_2 stretch. We can also exclude the presence in the experiment of the E9I-1 tautomer. First of all because E9I-1 would be obtained by means of double proton hopping from the more stable K9K-1 tautomer and if the latter is missing it is unlikely that the former is present. Then, because the highest frequency mode of E9I-1, the cytosine NH stretch at 3552 cm^{-1}, is too low in frequency to match the experimental signal at 3615 cm^{-1}. This is in spite of our QCT and DC-SCIVR frequencies being even blue shifted compared to the experiment, presumably due to the approximate description of hydrogen bonds and other interactions at the chosen affordable level of electronic theory."

(5) Likewise, on page 14, they exclude E9I-1 tautomer. How do they exclude the K9E-1 isomer, which has very a similar spectrum in Fig. 2 to that of K7E-1?

Authors' reply: We thank the Reviewer for this excellent point which was raised also by the other Reviewer. We copy here the same reply given to Reviewer 1.
We have now performed two new QCT calculations for the two tautomers (K7E-1 and K9E-1) involving the low frequency region. Just above 500 cm^{-1} (at 514 cm^{-1}), de Vries found in his experiment a single signal with significant intensity (J.M. Bakker, I. Compagnon, G. Meijer, G. von Helden, M. Kabelac, P. Hobza, and M.S. de Vries *Phys. Chem. Chem. Phys.* **6**, 2810 (2004)). This was assigned to a C-O-H in-plane bending. Based on scaled harmonic calculations de Vries found that if the K9E-1 tautomer were present, then a second signal just above 500 cm^{-1}, due to the guanine N-H out-of-plane bending, should be found. The corresponding guanine N-H out-of-plane bending signal for the K7E-1 tautomer was instead anticipated below 500 cm^{-1} meaning that if only the K7E-1 tautomer were present, then a single signal just above 500 cm^{-1} should be found, which is in agreement with the experiment. For this reason, de Vries concluded "we are tempted to assign the spectrum to structure D", D being the K7E-1 tautomer.
Our new QCT simulations confirm and strengthen de Vries' assignment because they anticipate the frequency of the guanine NH out-of-plane bending at 426 cm^{-1} for K7E-1 and at 509 cm^{-1} for K9E-1. Therefore, our conclusion is the same as de Vries and we rule out the presence of the K9E-1 tautomer.
In response to this comment we have inserted a new Figure in the supplementary information file. Furthermore, as suggested by the Reviewer, we have inserted the following new text when discussing the results:
"It remains to rule out the presence of K9E-1, which is lower in energy than K7E-1 and whose high frequency spectrum is basically indistinguishable from that of K7E-1. To this end we performed two QCT simulations (one

per tautomer) of the out-of-plane N-H bending because de Vries and co-workers could not assign any signal above 500 cm^{-1} in their experiments to this vibrational mode. The scaled harmonic calculations suggested for the out-of-plane N-H bending a frequency of 477 cm^{-1} for the K7E-1 tautomer, and a frequency of 508 cm^{-1} for the K9E-1 tautomer.(cit.) The missing peak just above 500 cm^{-1} allowed de Vries and co-workers to rule out the presence of K9E-1 in the experimental spectra. Our QCT simulations confirm and strengthen this conclusion estimating the target bending at 426 cm^{-1} for K7E-1 and at 509 cm^{-1} for K9E-1. Therefore, following de Vries' reasoning, we also rule out the presence of K9E-1 in the experimental spectra. A figure reporting the outcome of QCT calculations can be found in the Supplementary Information (see Figure SX)."
Also, we have modified the text at the beginning of page 17 in the following way:
"Furthermore, an examination of the low frequency region has allowed us to differentiate between the two tautomers and to rule out the presence of K9E-1 in the spectrum".

In particular, it would be nice to go slightly deeper into the discussion of points (4) and (5) to support more strongly the main conclusions of a very interesting and otherwise very clearly written manuscript.

Authors' reply: We think we have now addressed this comment in our revised manuscript.
